# Anti-Obesity Properties of a Novel Probiotic Strain of *Latilactobacillus sakei* CNTA 173 in *Caenorhabditis elegans*

**DOI:** 10.3390/ijms26073286

**Published:** 2025-04-01

**Authors:** Ignacio Goyache, Lorena Valdés-Varela, Raquel Virto, Miguel López-Yoldi, Noelia López-Giral, Ana Sánchez-Vicente, Fermín I. Milagro, Paula Aranaz

**Affiliations:** 1 Department of Nutrition, Food Science and Physiology, Faculty of Pharmacy and Nutrition, University of Navarra, 31008 Pamplona, Spain; igoyache@unav.es (I.G.); fmilagro@unav.es (F.I.M.); 2 Center for Nutrition Research, University of Navarra, c/Irunlarrea 1, 31008 Pamplona, Spain; mlyoldi@unav.es; 3 Centro Nacional de Tecnología y Seguridad Alimentaria (CNTA), Ctra. NA-134 Km.53, 31570 San Adrián, Spain; lvaldes@cnta.es (L.V.-V.); rvirto@cnta.es (R.V.); noelia22269@gmail.com (N.L.-G.); asanchez@cnta.es (A.S.-V.); 4 Navarra Institute for Health Research (IdiSNA), 31008 Pamplona, Spain; 5 Centro de Investigación Biomédica en Red de la Fisiopatología de la Obesidad y Nutrición (CIBEROBN), Instituto de Salud Carlos III, 28029 Madrid, Spain

**Keywords:** obesity, probiotics, *Caenorhabditis elegans*

## Abstract

Probiotic strains with health-promoting activities have emerged as a promising strategy to prevent or treat different metabolic syndrome-related disturbances, including obesity or type 2 diabetes. In this work, we characterize the probiotic properties of a novel strain of *Latilactobacillus sakei* (*L. sakei*) CNTA 173, and we demonstrate its anti-obesity properties using the in vivo model *Caenorhabditis elegans* (*C. elegans*). This new strain exhibited sensitivity to the entire spectrum of antibiotics analysed, gastric and intestinal in vitro resistance, β-galactosidase activity, and the ability to form biofilm and to produce acetic acid in vitro. Cell culture analyses demonstrated that *L. sakei* CNTA 173 was able to reduce the adhesion to Caco-2 cells of the pathogenic *E. coli* O157:H7 and to exert immunomodulatory capacity in RAW 264.7 and HT-29 in vitro models. Furthermore, supplementation with *L. sakei* CNTA 173 counteracted the deleterious effects of glucose in *C. elegans* by significantly reducing fat accumulation, enhancing the oxidative stress response, and extending lifespan by directly regulating the carbohydrate and lipid metabolism-related genes *acox-1*, *maoc-1,* and *daf-16*. Our results unveil new strain-specific mechanisms of action by which *L. sakei* CNTA 173 exerts beneficial effects in vitro and in *C. elegans*, and suggest potential application of this novel probiotic strain in the prevention and treatment of metabolic syndrome-related disturbances.

## 1. Introduction

The growing prevalence of metabolic-syndrome related diseases such as obesity and type 2 diabetes calls for new treatments and preventive strategies to slow down their evolution as well as in-depth knowledge of the factors involved in their development. In this context, the strong association between the gut microbiota and a healthy metabolic profile has led to the development of a new line of research on the possible use of certain probiotics as therapies to prevent or ameliorate these metabolic disorders [1].

Some of these probiotics, defined by the WHO as live microorganisms conferring health benefits to their hosts, have been shown to lessen some of the manifestations of these metabolic diseases, including the excess of fat accumulation, the dysregulation of blood glucose levels, and the development of low-grade inflammation [2,3,4]. Thus, specific bacterial strains, mainly lactic acid bacteria (LAB), have emerged as potential probiotic candidates against metabolic syndrome-related diseases due to their health-promoting properties [5,6], showing significant improvements as treatments in the most recent years [7].

Among the ancient genus *Lactobacillus*, for which numerous species have been described with anti-obesity properties, we find *Latilactobacillus sakei*, previously known as *Lactobacillus sakei*, a lacto-fermentative Gram-positive bacterium with the capability to colonize the gastrointestinal tract. Strains of this bacterium have already been reported to show metabolic improvements in human and animal trials, consolidating the *L. sakei* species as a promising treatment not only for obesity, but other metabolic syndrome-related diseases, including non-alcoholic fatty liver disease (NAFLD) and inflammation-related disturbances [8,9,10].

However, even though the knowledge around the beneficial effects of probiotics is rapidly growing, the underlying mechanisms of action of each specific strain are still not fully understood, thus opening a broad area of study [11,12]. In this line, the use of simple in vivo models such as *Caenorhabditis elegans* (*C. elegans*) represents a valuable advantage to investigate the potential health-promoting activities of different probiotic strains, including their ability to modulate carbohydrate and lipid metabolism, oxidative stress, or lifespan, together with deepening our understandingthe molecular mechanisms of action of each specific strain [13].

Here, we present a broad spectrum of in vitro tests to characterize the probiotic aptitude of a novel strain of *Latilactobacillus sakei*: *L. sakei* CNTA 173. Moreover, we investigate the health-promoting activities of *L. sakei* CNTA 173 in *C. elegans*, where we evaluate the effects of the probiotic supplementation on fat accumulation, oxidative stress, development, and lifespan. Finally, gene expression analyses of different metabolic pathways, including insulin/IGF-1 signalling (IIS) and fatty acid (FA) beta-oxidation, together with mutant strains of specific target genes, are performed to investigate the molecular mechanisms of action of *L. sakei* CNTA 173.

## 2. Results and Discussion

### 2.1. L. sakei CNTA 173 Identification, Growth, and Culture

Previous to the in vitro evaluation of the potential probiotic properties of *L. sakei* CNTA 173, the identification of our specific strain through genome sequencing was conducted. For this, all genomes classified as *Latilactobacillus sakei* available in the NCBI GenBank database (*n* = 73) were downloaded, and the ANI (Average Nucleotide Identity) index was calculated between these genomes and the genome of the strain *Latilactobacillus* sp. CNTA 173. The strain *Latilactobacillus* sp. CNTA 173 was identified as a member of the species *Latilactobacillus sakei*, and its current correct designation is *Latilactobacillus sakei* CNTA 173, which was deposited at the Spanish Collection of Type Cultures (CECT) under the accession number CECT 30971. The complete *L. sakei* CNTA 173 genomic sequence has been registered in NCBI with the reference number SUB15204130. The ANI value between the genome of strain CNTA 173 and the type strain genome of *Latilactobacillus sakei* was 98.73%, exceeding 96% (the threshold for species delineation) (Table S1). Moreover, the closest non-reference strains based on ANI were also *L. sakei* (Table S2).

To perform the phylogenetic analysis, the complete 16S rRNA gene sequence (1.28 Mbp) extracted from the strain’s genome was used (Table S3). According to the EzBioCloud results, the strain belongs to the genus *Latilactobacillus*, and the phylogenetically closest species are *Latilactobacillus sakei* subsp. sakei (100%), *Latilactobacillus sakei* subsp. carnosus (99.92%), and *Latilactobacillus curvatus* (99.36%).

Once the specific bacterial strain was identified, the first step to determine its probiotic potential was to characterize its in vitro growth parameters. To do tYhis, *L. sakei* CNTA 173 was cultured anaerobically in the most common growth medium used for LAB (MRS) at 30 °C for 48 h. At predefined time intervals, samples were collected, and microbial counts were obtained by using the plate count method. Figure S1 shows the fit of the Gompertz model [14] to the experimental values of the growth curve.

As shown in Table S4, both fits were good (R value of 0.99, RMSE value of 0.11). Values of A, B, C, and M allowed us to compare the growth kinetic of this strain with other strains of different species.

### 2.2. The In Vitro Characterization of L. sakei CNTA 173 Demonstrates Its Suitability as a Probiotic Strain

Prior to the in vivo functional assays, in vitro determinations were carried out to determine the suitability of the *L. sakei* CNTA 173 strain as a possible probiotic. To this end, different probiotic characterization tests described in the Materials and Methods section were carried out and are listed below.

One of the fundamental tests is the determination of the strain sensitivity to antibiotics. Microbial feed additives should not add to the pool of antimicrobial resistance (AMR) genes already present in the gut bacterial population or otherwise increase their spread [15]. Thus, the minimal inhibitory concentrations (MICs) of different antibiotics were determined by the broth microdilution method, and results were interpreted using the breakpoints recommended by the EFSA [15]. As detailed in Table 1, the strain *L. sakei* CNTA 173 was susceptible to ampicillin, chloramphenicol, clindamycin, erythromycin, gentamycin, kanamycin, streptomycin, and tetracycline, according to the MIC breakpoint values for *Lactobacillus* facultative heterofermentative. Therefore, *L. sakei* CNTA 173 is considered suitable for use as a microbial feed additive, according to EFSA.

**Figure 1:**
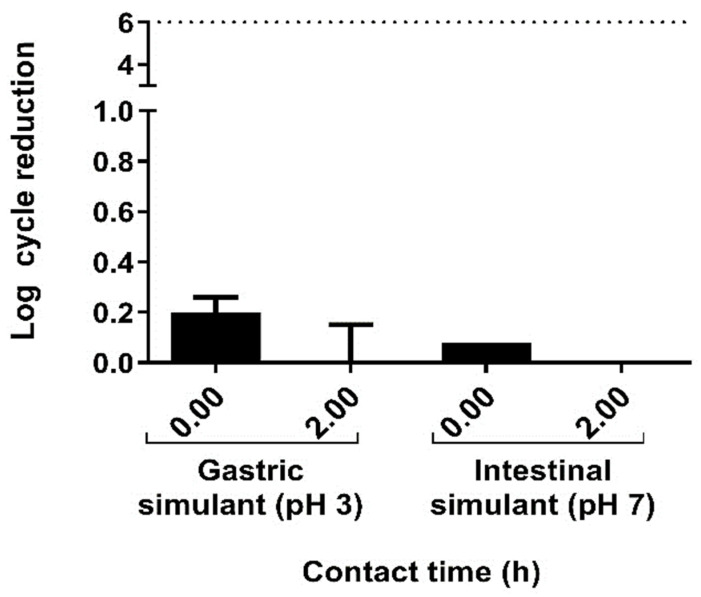
Gastric and intestinal fluids tolerance of *L. sakei* CNTA 173 (log cycle reduction).

It has been described that, to confer health benefits, a probiotic supplement must reach the ileum in a minimum concentration of 6 log CFU/g or CFU/mL [16]. Thus, potential probiotic bacteria must survive the gastrointestinal tract conditions in order to reach the colon environment in adequate numbers. In this sense, we observed that *L. sakei* CNTA 173 showed high resistance to simulated gastric fluid (Figure 1). This strain retained its viability after 2 h, and the viable count showed a decrease of less than 0.1 log cycles with respect to the initial cell concentration. Moreover, after 2 h exposure to simulated intestinal fluid, this strain displayed good survival capacity, since the cell population remained stable in 7 log CFU/mL, demonstrating that *L. sakei* CNTA 173 exhibits good adaptation to simulated gastric and intestinal fluids.

The beneficial clinical effects of certain LABs have been attributed to their ability to produce SCFAs, which play a very important role in maintaining intestinal and immune homeostasis in the human body [17]. In this study, the content of the most abundant SCFAs (acetate, propionate, and butyrate) were analysed by GC-MS in different culture media of *L. sakei* CNTA 173. As shown in Table 2, *L. sakei* CNTA 173 was able to produce acetic acid at 48 h with a carbohydrate source (glucose, Synergy 1, and P95), and without adding a carbon source, ranging from 0.40 g/L to 1.46 g/L. Moreover, propionic acid was quantified in the medium without adding a carbon source (14.17 ± 5.20 mg/L). Butyric acid was not detected in any medium. Therefore, *L. sakei* CNTA 173 could produce acetic acid in all cases tested, as previously observed in another strain of the same species [18]. Acetate has been reported to play an important regulatory role in body weight control and glucose homeostasis [19].

**Figure 2:**
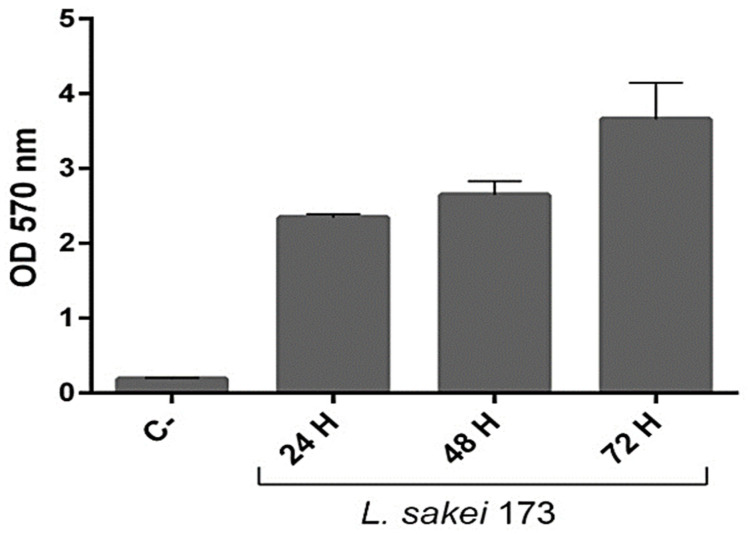
Biofilm property of the *L. sakei* CNTA 173. Data are presented as mean ±  SD of three independent experiments, each performed in triplicate.

Successful colonization relies on the selective adhesion of bacteria to specific epithelia, such as the intestinal mucosa, effectively extending and stabilizing their residence within the epithelial lining. This adhesion mechanism is allowed by the ability of probiotics to form communities called biofilms, which represents an advantage for the growth of microbial populations in the presence of diverse abiotic or biotic factors [20] and aids in the exclusion of pathogenic bacteria through competitive inhibition or steric hindrance while also potentially activating the immune response of the host cell [21]. Interestingly, we demonstrated that *L. sakei* CNTA 173 exhibited a high capacity to form a biofilm (Figure 2), as indicated by the absorbance values obtained, all higher than 0.5, ranging from 2 ±  00 (24 h) to 3.65  ±  0.48 (72 h), with significant differences (*p*  <  0.05) between the isolates.

β-galactosidase is a key enzyme in the lactose metabolism process and is produced by bacteria that can utilize lactose as an energy source. When a bacterium exhibits β-galactosidase activity, it means that it can produce and secrete this enzyme to break down lactose into glucose and galactose. This enzymatic activity is important in the identification and characterization of certain bacteria, but also in applications such as the dairy industry [22]. Thus, we tested the production of β-galactosidase by *L. sakei* CNTA 173 throughout the qualitative in vitro test by applying sterile filter paper disks impregnated with ONPG. The result (Figure S2) exhibited the colour change after 5 h of exposure to ONPG, demonstrating that *L. sakei* CNTA 173 has β-galactosidase activity, and thus, is able to break down the disaccharide lactose into its monosaccharide components, glucose and galactose. Finally, we investigated whether *L. sakei* CNTA 173 shows bile salt hydrolase (BSH) activity using qualitative direct plate assay. Probiotic *lactobacilli* with bile salt hydrolase activity have been observed to have a higher possibility of survival in the gastrointestinal tract. Furthermore, it has been described that probiotics with BSH activity show cholesterol-lowering activity in vivo [23]. However, this strain produced similar colony types on plates with or without tauro-conjugated and glyco-conjugated bile salts (without a halo around the microbial growth in media supplemented with bile salts). In addition, its growth was lower in the media supplemented with bile salts and even inhibited in the medium with sodium salt of glycodeoxycholic (Figure S3). Therefore, *L. sakei* CNTA 173 did not show the ability to hydrolyse tauro-conjugated and glyco-conjugated bile salts.

### 2.3. L. sakei CNTA 173 Reduces the Adhesion of the Pathogenic E. coli O157:H7 In Vitro

**Figure 3:**
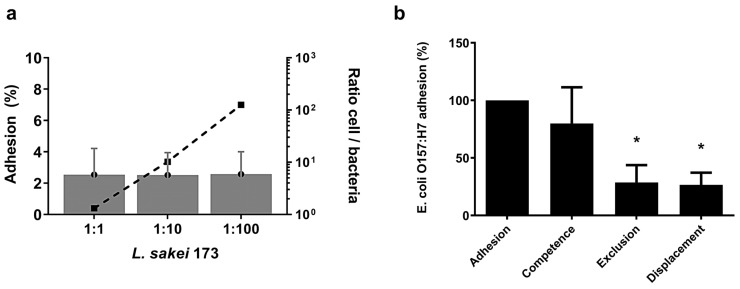
In vitro adhesion studies. (**a**) Effect of bacterial concentration on the adhesion of *L. sakei* to Caco-2 cells. The data presented are the means of three independent assays with three wells per assay. (**b**) Adhesion of *E. coli* on Caco-2 cells in competence with *L. sakei* (*L. sakei* + *E. coli*), exclusion (*L. sakei/E. coli*), or displacement (*E. coli*/*L. sakei*). *L. sakei* was added at a concentration of 2.6 × 10^6^ ± 1.1 × 10^6^ CFU/well and *E. coli* at 9.2 × 10^6^ ± 6.5 × 10^6^ CFU/well for the different experiments. The data presented are the means of three independent assays with three wells per assay. * Indicates the means, which were significantly different (*p* < 0.05) from the control data for adhesion of *E. coli* alone.

We then evaluated the impact of *L. sakei* CNTA 173 on adhesion of the pathogenic bacteria *E. coli* O157:H7, using the cellular model Caco-2. Since this cell line exhibits similar characteristics to a mature enterocyte [24], Caco-2 cells have been reported to mimic the in vivo conditions of adhesion and infection of pathogenic bacteria [25]. For this purpose, we initially determined the proper ratio of cells/bacteria and their dynamics. Figure 3a shows the adhesion of *L. sakei* to Caco-2 cells depending on the cell/bacteria ratios 1:3, 10:2, and 125:3. Adhesion was 2.5% in the three different conditions studied, so no significant differences in adhesion were observed between the different cell/bacteria ratios evaluated.

The displacement of *E. coli* by *Lactobacillus* GG or by *Lacticaseibacillus casei* Shirota and vice versa was previously reported as well, when both strains were applied at similar concentrations. However, the concentration-dependent kinetics of adhesion is a usual phenomenon [25]. Bogovic et al. [26] showed, for the *Lactobacillus gasseri* K7 strain, that the saturation concentration was about 3.5 × 10^8^ CFU/well. In this research, the maximum number of added bacteria for *L. sakei* was 1.8 × 10^8^ ± 9.1 × 10^7^ CFU/well.

Then, for the ratio cells/bacteria 1:10, the ability of *L. sakei* CNTA 173 to reduce *E. coli* adhesion on Caco-2 cells was investigated. The percentage of adhesion of *E. coli* in competence, displacement, and exclusion assays, respectively, of *E. coli* adhesion when this bacterium was alone in the culture is shown in Figure 3b. *L. sakei* was added at a concentration of 2.6 × 10^6^ ± 1.1 × 10^6^ CFU/well and *E. coli* at 9.2 × 10^6^ ± 6.5 × 10^6^ CFU/well for the different experiments. When Caco-2 cells were previously exposed to *L. sakei* in exclusion assays, *E. coli* adhesion was reduced to 71%. Also, the reduction in *E. coli* adhesion by *L. sakei* in displacement assays was about 73%. However, when *E. coli* was incubated simultaneously with *L. sakei* (*L. sakei* + *E. coli*, competition assays), no significant differences were observed regarding individual bacterial adhesion to Caco-2 cells (Figure 3b).

In our study, *L. sakei* decreased *E. coli* adhesion to epithelial cells when Caco-2 cells were exposed either before or after the pathogen. Other strains of *lactobacilli* were previously shown to have similar effects. Todoriki et al. [27] tested the ability of 12 *lactobacilli* strains belonging to 6 species of the *Lactobacillus acidophilus* group to inhibit the adhesion of enterotoxigenic *E. coli* (ETEC). Studies of another two *L. acidophilus* strains [28,29] showed similar inhibition of ETEC strain adhesion. The proposed mechanisms involved in this protection against pathogen adhesion are either non-specific steric hindrance of receptors for pathogens, preventing them from accessing the cell surface of enterocytes, or the effect of substances present in the supernatant of the *lactobacilli*. Also, bifidobacteria have been previously reported to inhibit the adhesion of other several Gram-negative pathogens such as ETEC, enteropathogenic *E. coli* (EPEC), and *Salmonella typhimurium* [30]. Gagnon et al. [31] also observed an important reduction in *E. coli* O157:H7 adhesion in competence, exclusion, or displacement assays in the presence of bifidobacteria.In this work, we have also observed that *L. sakei* CNTA 173 reduces *E. coli* O157:H7 adhesion in exclusion or displacement assays, but not in competition assays. *E. coli*. However, it needs to be considered that the dynamics of probiotic–bacteria interactions are complex and strain-specific. In fact, Lee et al. 2003 [24] showed that different strains of *lactobacilli* displayed uneven affinities for *E. coli,* and the reported individual effects of *lactobacilli* were absent when exposed in competition. Overall, the different in vitro assay conditions (pH, well size, incubation medium, incubation time, cell lines, etc.) makes difficult the comparison of our results with those reported in the literature.

### 2.4. L. sakei CNTA 173 Exhibits Immunomodulatory Capacity In Vitro

Different strains of *L. sakei* have been previously shown to increase the secretion of several cytokines [9], and it has been suggested that such immunomodulation would be beneficial in fighting against pathogenic infections. To address a possible effect of *L. sakei* CNTA 173 in modulating cytokine production, the murine macrophage cell line RAW 264.7 and the human intestinal cell line HT-29 were used as cellular models for inflammation, where we investigated the levels of IL-6, IL-10, IL-8, and TNF-α after exposure to LPS. As a positive control for anti-inflammatory activity, hydrocortisone was used at a concentration of 50 µM prior to LPS treatment for the RAW 264.7 cells and 100 µM for the HT-29 cell line.

**Figure 4:**
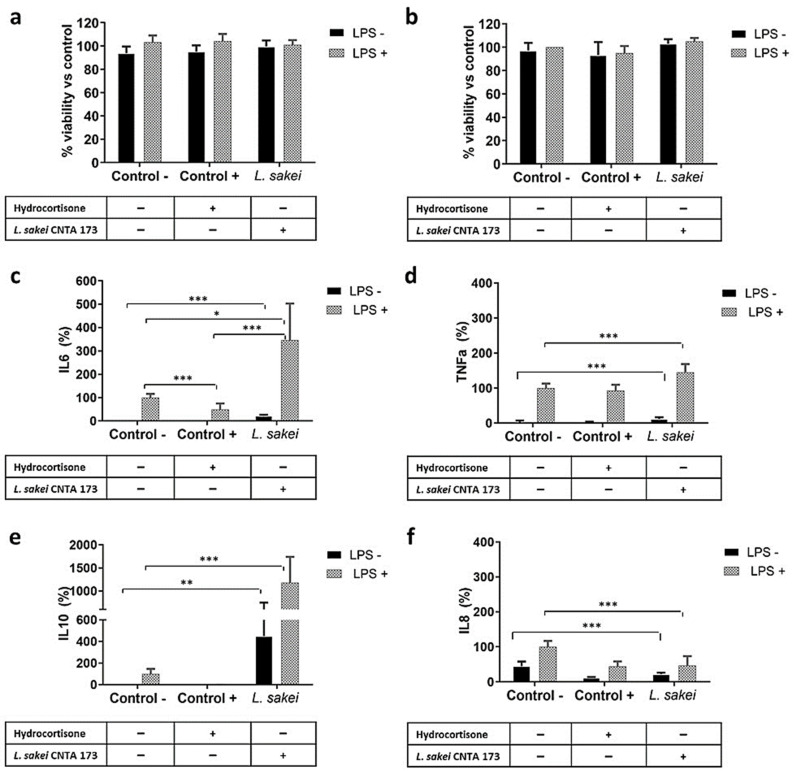
In vitro evaluation of the immunomodulatory activity of *L. sakei* CNTA 173. Assessment of cell viability after hydrocortisone treatment or *L. sakei* in untreated or LPS-stimulated (**a**) RAW 264.7 (**b**) and HT-29 cells. (**c**) Effect on the production of IL-6 of LPS-stimulated RAW 264.7 cells. (**d**) Effect on the production of TNF-α of LPS-stimulated RAW 264.7 cells. (**e**) Effect on the production of IL-10 of LPS-stimulated RAW 264.7 cells. (**f**) Effect of *L. sakei* on IL-8 production by HT-29 cells. HT-29 cells were treated with *L. sakei* pellet for 3 h, and then stimulated with LPS for 18 h. IL-8 production was quantified by ELISA. Statistics were performed as described in the Materials and Methods section. One-way ANOVA followed by Dunnet’s comparison was applied to (**a**,**b**,**d**–**f**); Kruskal–Wallis for (**c**). Data represent the mean ± SD of three independent experiments with three technical replicates each. * *p* ≤ 0.05, ** *p* ≤ 0.01, *** *p* ≤ 0.001.

We initially investigated whether *L. sakei* CNTA 173, with or without the presence of LPS, could affect the viability of RAW 264.7 and HT-29 cells, as this could distort the effect on cytokine production. As depicted in Figure 4a, RAW 264.7 cell viability was not altered by the treatment with hydrocortisone or *L. sakei* in normal or LPS-stimulated cells, assuring that the differences in cytokine levels are directly due to differences in their synthesis and not variations in cellular density. In a similar manner to RAW 264.7, HT-29 viability was not affected by the presence of *L. sakei* CNTA 173, assuring that the cell number is consistent among all the conditions (Figure 4b).

As observed in Figure 4, under basal conditions (without LPS stimulation), *L. sakei* CNTA 173 significantly modified the production of IL-6 (Figure 4c), TNF-α (Figure 4d), and IL-10 (Figure 4e) by RAW 264.7 macrophages. Furthermore, when cells were pre-treated with *L. sakei* for 3 h and then stimulated with LPS, the secretion of these three cytokines drastically increased in comparison with the LPS control cells. To investigate whether *L sakei* CNTA 173 had an effect modulating IL-8 production in intestinal cells, HT-29 cells were incubated with the bacterial pellet for 3 h followed by stimulation with LPS for 18 h. In non-stimulated cells, *L. sakei* CNTA 173 did significantly decrease IL-8 secretion, and this effect was maintained in LPS-stimulated cells, similarly to the effect of the positive control, hydrocortisone (Figure 4f).

It is known that liposaccharides and other proteins present in the cellular wall of *L. sakei* bacteria can interact with macrophage receptors, stimulating cytokine production. In our study, *L. sakei* CNTA 173 modulated the cytokine secretion in RAW 264.7 and HT-29 cells, both under non-inflammatory and inflammatory conditions. It has been shown previously that some strains of *Lactiplantibacillus plantarum* and *L. sakei* can stimulate the production of cytokines in immunosuppression models. According to our results, potentially, *L. sakei* CNTA 173 could be used as an immune-stimulating probiotic for patients suffering from immunosuppressive conditions.

Interestingly, in intestinal HT-29 cells, *L. sakei* CNTA 173 reduced the production of IL-8 in non-stimulated and stimulated cells. Several *lactobacilli* species are resident bacteria in the human intestine and have important roles in modulating immune responses [32].The interactions among the different bacteria present in the intestine lead to various immune scenarios, highlighting the importance of diversity in human microbiota composition. Other studies have demonstrated that *Ligilactobacillus salivarius* and *L. plantarum* can reduce the production of IL-8 in human gut cells in pro-inflammatory conditions [33]. This evidence suggests that *lactobacilli* strains could be useful in the treatment of inflammatory gut diseases. Our results support this hypothesis, specifically with *L. sakei* CNTA 173.

### 2.5. L. sakei CNTA 173 Reduces Fat Accumulation, Enhances Oxidative Stress Response, and Increases Lifespan in C. elegans

*C. elegans* is a microscopic nematode that has become a highly popular animal model in the study of many research fields, such as antimicrobial drug discovery, neurology, embryo development, and genetic variations [34,35,36,37]. In the case of obesity and other metabolic-related diseases, this nematode constitutes a powerful in vivo model to screen the physiological and molecular effects of different bioactive compounds, including probiotics, with potential applications regarding the metabolic aberrations of these diseases. *C. elegans* stores fat in lipid droplets, which can be quantified by microscopy after staining with fat-soluble dyes such as Nile red. Additionally, the gene ontology regarding metabolism-related genes places this model as an ideal starting point to further understand how probiotics can affect lipid metabolism and fat accumulation [38]. Here, we investigate the potential anti-obesity properties of *L. sakei* CNTA 173 using *C. elegans*.

**Figure 5:**
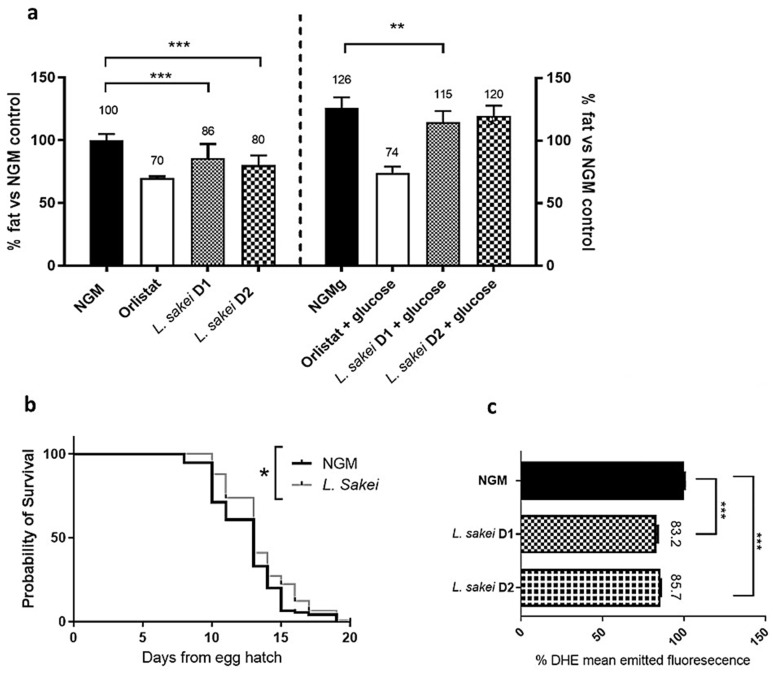
*L. sakei* CNTA 173 improves health markers and reduces fat accumulation in *C. elegans*. (**a**) Fluorescence microscope study of Nile red-stained worms in NGM or NGMg media, supplemented with *L. sakei* in two different dosages (D1: 3 × 10^10^ UFCs; D2: 3 × 10^9^ UFCs) vs. a control group. Results are expressed as the mean ± standard deviation relative to control worms in an NGM medium or in a glucose-loaded (10 mM) NGM medium. Significance refers to the effect of *L. sakei* with respect to control worms in an NGM medium or in a glucose-loaded (10 mM) NGM medium (Student’s *t*-test, ** *p* < 0.01; *** *p* < 0.001). (**b**) *L. sakei* increases the lifespan of *C. elegans.* The results were plotted in a survival curve chart, with significance referring to the effect of *L. sakei* on the total number of alive worms for longer period (log-rank Mantel–Cox test, * *p* < 0.05). (**c**) ROS production quantification measured by mean emitted fluorescence by the ROS-binding molecule DHE on *L. sakei*-treated worms (D1: 3 × 10^5^ UFCs; D2: 3 × 10^6^ UFCs) against NGM media control group. Results are repressed as the mean ± standard deviation relative to the control group, with significant differences found after performing statistic studies (ANOVA test and Tukey’s multiple comparison test, *** *p* < 0.001).

First, we demonstrated that different doses (D1: 3 × 10^5^ CFU and D2: 3 × 10^6^ CFU) of *L. sakei* CNTA 173 induced lipid-reducing activity in N2 wild-type worms, both in NGM and NGMg plates (Figure 5a). Specifically, we observed that worms grown in the presence of the probiotic accumulated 9.2% less fat than the control group in the NGMg plates, augmenting to 20% less fat in the NGM plates. Interestingly, this lipid-reducing effect did not affect the correct development of the worms, with both the probiotic and control group plates exhibiting the presence of eggs and L1 larvae with no differences at the time of appearance (Figure S4). These results go along with previous anti-obesity properties described for other *L. sakei* strains. For example, Won et al. described that supplementation with the *L. sakei* ADM14 strain reduced by 25.8% (*p* < 0.05) body weight gain in diet-induced obese mice in comparison with the non-supplemented high-fat diet group [10]. Along the same line, other probiotic strains have been reported to reduce the fat content of *C. elegans*, including *Bifidobacterium*, *Pediococcus*, and other *Lactobacillus* strains [13]. However, to our knowledge, this is the first time describing the fat-reducing activities of an *L. sakei* strain in *C. elegans*.

As a simple model organism, *C. elegans* lipid metabolism relates to different physiological processes, including oxidative stress, aging, and lifespan. Thus, once we demonstrated the lipid-reducing activity of *L. sakei* CNTA 173 in *C. elegans*, we analysed other health span-related parameters, such as life expectancy and oxidative stress. Thus, we observed that supplementation with *L. sakei* CNTA 173 induced a significant extension of the nematode’s lifespan in comparison with NGM control plates (*p* = 0.011, Figure 5b). This effect on the nematode’s lifespan is consistent with the results described in previous works, where different strains of probiotics have been shown to exert similar effects [39,40,41]. In the case of *L. sakei* species, two works had previously reported health-promoting activities in this nematode model, including *L. sakei* 20D49 [42] and LFR20-007 [43], which significantly improved the *C. elegans* lifespan. However, and contrary to our experiment, most studies have evaluated the effect of *L. sakei* over the nematode’s lifespan as the sole food source in comparison with the standard OP50 diet. This replacement could imply a lower energy density and changes in the lipid composition per se, which in turn might affect *C. elegans* lifespan in a probiotic-independent manner.

Some probiotics, mainly other *Lactobacilli* and *Bifidobacteria*, and other bioactive molecules such as postbiotics, have already been demonstrated to improve the oxidative stress response [41,44]. Oxidative stress is a consequence of an imbalance between the production and accumulation of ROS or the failure of a biological system to detoxify these reactive products [45]. For this reason and based on the effect on fat accumulation and the lifespan of the worm, we decided to evaluate how *L. sakei* CNTA 173 could be affecting oxidative stress, a process closely related to metabolic diseases. For these experiments, glucose-loaded NGM plates were used to ensure a high-stress condition that could trigger the oxidative stress and aging response. Quantification of ROS accumulation through the ROS-binding fluorescent dye DHE revealed that supplementation with both doses of *L. sakei* CNTA 173 in a glucose-loaded medium (NGMg plates) from L1 to L4 stage significantly reduced the accumulation of ROS by 14 to 17% when compared with control worms (Figure 5c). Similarly to the result with the fat-reducing activity, this is the first work describing the antioxidant ability of a probiotic strain of *L. sakei* in *C. elegans*. However, while *C. elegans* is accepted as a powerful screening model due to the previously mentioned strengths, its limitations must be acknowledged. Some of the key mammalian physiological features, such as adaptive immunity, circulatory system, and complex organ structures, are not found in this initial model, and thus, the extrapolation of these results could be affected. Hence, future research should be conducted on murine or higher mammalian systems to further investigate the properties and potentials of the *L. sakei* CNTA 173 probiotic strain.

In summary, we found that the novel strain *L. sakei* CNTA 173 was able to reduce fat accumulation in *C. elegans*, both in NGM and gNGM medium, while enhancing ROS response and the lifespan of this nematode.

### 2.6. L. sakei CNTA 173 Modulates Lipid and Carbohydrate Metabolism in C. elegans

To determine the molecular mechanisms underlying the physiological effects of *L. sakei* CNTA 173 on *C. elegans*, we investigated how this bacterium was able to modulate the expression of key genes involved in fatty acid synthesis and degradation together with the insulin signalling IIS pathway.

**Figure 6:**
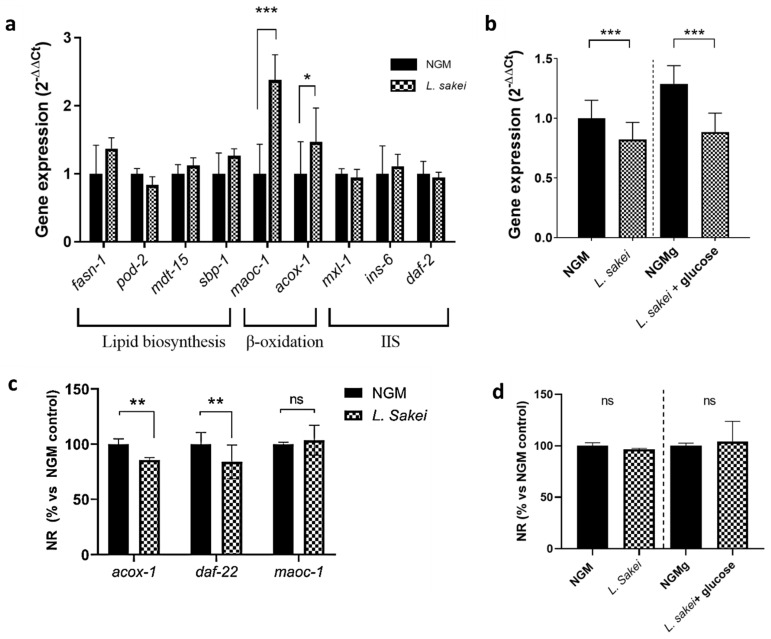
*L. sakei* CNTA 173 modulates the expression of key metabolism-related genes in *C. elegans.* (**a**) Gene expression analysis quantified by real-time PCR (qPCR) using the 2^−∆∆Ct^ method in *C. elegans*. Gene expression levels were normalized to the housekeeping gene (*pmp-3*). Data are expressed using the 2^−∆∆Ct^ method. A two-way ANOVA (main effects: *L. sakei*, glucose, and their interaction) was carried out to evaluate statistical differences between groups. Two-way ANOVA results when *L. sakei* factor is significant: * *p* < 0.05; *** *p* < 0.001. (**b**) *Daf-16* gene expression analysis quantified by real-time PCR (qPCR) in *C. elegans* grown under normal conditions. Gene expression levels were normalized to the housekeeping gene (*pmp-3*). Data are expressed using the 2^−∆∆Ct^ method. Differences in expression levels were studied by Student’s *t*-test (*p* < 0.05; ***). (**c**) Nile red quantification of *L. sakei*-treated worms in *acox-1* mutant, *daf-22* mutant, and *maoc-1* mutant in an NGM medium. Statistical analyses were performed using Student’s *t*-test to study the differences between groups; ** *p* < 0.01; ns not significant. (**d**) Nile red quantification of *L. sakei*-treated worms in *daf-16* mutant in an NGM medium and in a glucose-loaded (10 mM) NGMg medium. Statistical analyses were performed using Student’s *t*-test to study differences between groups (ns not significant).

No differences were observed in the expression of the lipogenesis-related genes *sbp-1* (ortholog of human *SREBF*), *fasn-1* (ortholog of human *FAS*), *mdt-15* (ortholog of human *MED15*), *mxl-3* (ortholog of human *MAX*), and *pod-2* (ortholog of human *ACC*) [38] between *L. sakei* CNTA 173-cultured and control worms (Figure 6a), suggesting that the lipid-reducing activity of this probiotic was not attributed to a reduced fatty acid synthesis. However, when cultured in the presence of *L. sakei,* the nematodes overexpressed *acox-1* (ortholog of human *ACOX1*) and *maoc-1* (ortholog of human *HSD17B4*), two key genes in the peroxisomal beta-oxidation of fatty acids. Its overexpression suggests a higher degradation rate of the fatty acids induced by *L. sakei* CNTA 173, therefore explaining the reduction in total fat accumulation.

Due to the important role of the insulin signalling pathway (IIS) in the worm’s metabolism, but also in the regulation of the nematode’s growth and lifespan, we analysed the gene expression levels of two key genes of this pathway: *daf-2* and *daf-16*. In *C. elegans*, IIS is regulated by insulin-like peptide ligands that bind to the insulin/IGF-1 transmembrane receptor (IGFR) ortholog *daf-2*. Daf-2/IGFR controls the activity of a conserved phosphoinositide 3-kinase (PI3K)/Akt kinase cascade, culminating in the regulation of a FoxO transcription factor ortholog: *daf-16* [46]. Due to the simplicity of the nematode, the IIS pathway is not only involved in the metabolism of sugars, but also in stress response signalling (oxidative stress, hypoxia, and heat stress), aging, and fat metabolism [47]. Although no changes were observed in *daf-2* gene expression (Figure 6a), *L. sakei* CNTA 173 induced a significant downregulation of *daf-16* in both normal and glucose-loaded conditions, counteracting the upregulating effect of glucose (Figure 6b). A similar result was observed by Kent and colleagues, who demonstrated an overall overexpression of all isoforms of *daf-16* in the presence of glucose (25 mM) in N2 worms without changes in *daf-2* expression.

To confirm our findings, we investigated whether the fat-reducing activity of *L. sakei* CNTA 173 was maintained in specific strains with mutations for both the beta-oxidation and IIS signalling pathways (*acox-1*, *maoc-1*, *daf-22*) and in the IIS signalling modulator *daf-16*. Nile red staining showed that *acox-1* and *daf-22* mutations did not prevent the *L. sakei*-mediated reduction in fat accumulation (Figure 6c). However, probiotic-treated *maoc-1*-mutant worms did not show a significant reduction in body fat accumulation, suggesting the need of this gene to maintain the fat-reducing activity of this probiotic. These results further confirm that *L. sakei* CNTA 173 can affect the expression levels of beta-oxidation pathway-related genes, thus modulating lipid storage.

Interestingly, *daf-16* mutants showed no significant body fat effect after exposure to *L. sakei* CNTA 173 in both normal and high-glucose conditions (Figure 6d), demonstrating that the effect of *L. sakei* CNTA 173 is *daf-16* dependent. This suggests that the effect on the IIS pathway is accomplished at the transcription factor level, since other genes related to upstream effector proteins were not altered by *L. sakei* CNTA 173.

Thus, while the higher content of glucose in the media upregulates *daf-16*, the presence of our probiotic seems to activate fatty acid degradation though the beta-oxidation pathway, possibly activating a signalling cascade and causing a decrease in glucose uptake from the medium. These results are similar to the ones described by Martorell et al. in their studies on *Bifidobacterium animalis* subsp. *lactis* CECT 8145 and its effects on a *C. elegans* model [48]. Similarly to the effect of our probiotic, their study demonstrated the ability of *B. animalis* subsp. *lactis* CECT 8145 to improve physiological parameters through modulation of the beta-oxidation pathway, increasing the expression of *acox-1*. In a later publication, they also described a lipid accumulation-lowering effect by the same probiotic and one of its metabolites, lipoteichoic acid, the effect being dependent on the *daf-16* gene expression like in our case [49]. Furthermore, other authors, like Mingkun Gu et al. [50], have also attributed the beneficial health effects of *Lactobacillus pentosus* MJM60383, such as limiting lipid accumulation, to its ability to activate the beta-oxidation pathway. Finally, other probiotic strains, including *Pediococcus acidilactici* CECT 9897, have been shown to ameliorate the effects of glucose exposure by enhancing this lipid catabolic process [47].

In conclusion, *L. sakei* CNTA 173 can regulate the fatty acid and the carbohydrate metabolism pathways in *C. elegans* by increasing the expression of peroxisomal beta-oxidation-related genes, including *maoc-1*, and by modulating *daf-16* gene expression, which would contribute to explain the anti-obesity and health-promoting activities of this probiotic.

## 3. Materials and Methods

### 3.1. Bacterial Strain and Growth Conditions

The strain used in this study was *L. sakei* CNTA 173, which was derived from CNTA culture collection. This strain was collected from pea by-products.

#### 3.1.1. Growth Conditions

The bacterium was grown in MRS broth (Merck KGaA, Darmstadt, Germany) at 30 °C under anaerobic conditions. The bacterial stock, kept at −80 °C in cryovial (Scharlab S.L., Barcelona, Spain), were spread onto the surface of MRS agar and incubated for 2 days. A single colony was picked to inoculate 10 mL MRS broth, which, after 19 h incubation, was used to inoculate bottles of 50 mL fresh MRS broth (10^6^ CFU/mL). The tubes with inoculated media were incubated under anaerobic conditions at 30 °C for 48 h. At the defined sampling points, serial decimal dilutions were prepared, and the number of cells was determined by the plate count method with the use of MRS agar. Plates were incubated in anaerobic chamber at 37 °C for 48 h, and colonies were enumerated. Microbial counts were reported as log CFU/mL. Culture pH was measured with a pH meter Basic 20+ (Crison Instruments S. A., Barcelona, Spain).

#### 3.1.2. Growth Kinetic Determination

The kinetic data were modelled using the online tool Python 3.9 (Python Software Foundation, Python Language Reference, version 3.9.13) according to the four-parameter Gompertz model. This software was used to establish the initial bacteria count (A) (log CFU/mL), maximum growth rate (B) ([hours^−1^]), difference between the initial bacteria count and the maximum bacteria count (C) (log CFU/mL), time required by microorganism to achieve the maximum growth rate (M) (hours), and the lag time (LPD) (hours) for *L. sakei* CNTA 173 cultivated in MRS [14].

### 3.2. Assessment of Antibiotic Resistance

Antibiotic resistance of the potential probiotic was assessed according to the ISO 10932/IDF 223 standard (2010) [51]. Minimal inhibitory concentrations (MICs) of ampicillin, chloramphenicol, clindamycin, erythromycin, gentamycin, kanamycin, streptomycin, and tetracycline (Sigma-Aldrich, St. Louis, MO, USA) were determined using the microdilution method on a 96-well microtiter plate using susceptibility test media. Minimal inhibitory concentrations (MICs) of the antibiotics were determined as the smallest concentration of antibiotic that prevented visible microbial growth. Based on MICs, the strain was designated as resistant or susceptible following the breakpoints and guidelines provided by the European Food Safety Authority (EFSA) [15]. The MIC values were calculated in three independent experiments.

### 3.3. Resistance to Simulated Gastrointestinal Conditions

Simulated gastric and intestinal fluids were prepared according to INFOGEST in vitro digestion method [52]. The *L. sakei* CNTA 173 suspension in early stationary phase was centrifuged (2348× *g*/10 min). The pellet was washed with phosphate-buffered saline (PBS), and a count was made in a Thoma Chamber to obtain a suspension at a concentration of 2 × 10^7^ CFU/mL. This suspension was dispensed into 4 microtubes at a rate of 1 mL per microtube (2 microtubes for studying resistance to gastric simulant (0 and 2 h) and 2 microtubes for studying resistance to intestinal simulant (0 and 2 h)). The four microtubes were centrifuged (21,130× *g*/10 min), and the pellets obtained were resuspended in 1 mL of gastric simulant and incubated at 37 °C in an orbital shaker (150 rpm) until the moment of sampling. Two microtubes were taken at 0 and 2 h, centrifuged (21,130× *g*/10 min), washed with PBS, and viable counts were determined on MRS agar plates. The remaining microtubes (2 microtubes: 0 and 2 h of the study of resistance to intestinal simulant) were centrifuged (21,130× *g*, 10 min), and the pellet obtained was resuspended in 1 mL of intestinal simulant and incubated at 37 °C in an orbital shaker (150 rpm) until the moment of sampling.

### 3.4. Ability of the Potential Probiotics to Produce Short-Chain Fatty Acids (SCFAs)

Uncontrolled-pH batch cultures were performed in a defined medium with the following compositions used: proteose peptone (10 g/L) (Sigma-Aldrich), beef extract (10 g/L) (Sigma-Aldrich), yeast extract (5 g/L) (Scharlau, Barcelona, Spain), polysorbate 80 (1 mL/L) (Scharlau, Spain), ammonium citrate (2 g/L) (Sigma-Aldrich), magnesium sulphate (0.2 g/L) (Panreac Applichem, Barcelona, Spain), manganese sulphate (0.056 g/L) (Panreac), and dipotassium phosphate (2 g/L) (Panreac). The media were added with 2% (*w*/*v*) of two different commercial prebiotic substrates: Synergy 1 (Beneo-Orafti NV, Tienen, Belgium) and P95 (Beneo-Orafti), or glucose (Sigma-Aldrich) as positive control. Each medium was distributed into individual tubes. Additionally, an extra tube without a carbon source was included as a negative control. Then, tubes were inoculated with *L. sakei* CNTA 173 at a final level of about 106 CFU/mL. Fermentations were carried out in anaerobic conditions at 30 °C for 48 h. Samples were taken at 0 and 48 h for quantification of SCFAs by GC-MS, as described previously [53]. Then, 50 mL of each culture collected at the defined sampling points was centrifuged (4500× *g* for 10 min) to obtain supernatants, which were immediately frozen at −20 °C until their use. These cell-free supernatants were filtered through 0.2 µm pore-size filters. All cultures were carried out in triplicate.

### 3.5. Assessment of β-Galactosidase Activity

The β-galactosidase activity of *L. sakei* CNTA 173 was assessed employing sterile filter paper disks impregnated with o-nitrophenyl-β-D-galactopyranose (ONPG disks, Fluka, Buchs, Switzerland), according to the manufacturer’s instructions and as previously described by dos Santos et al. 2015 [54].

The bacterial stock, kept at −80 °C in cryovial (Scharlab S.L., Barcelona, Spain), were spread onto the surface of agar-MRS and incubated anaerobically (anaerobic chamber MG500: Don Whitley Scientific, Yorkshire, UK) at 37 °C for 48 h. A colony of *L. sakei* CNTA 173 was picked and emulsified in a tube containing an ONPG disk soaked with 0.1 mL of sterile 0.85% (*w*/*v*) sodium chloride solution (PBS). The tubes were incubated at 30 °C and checked for up to 24 h. The release of a yellow chromogenic compound, o-nitrophenol, served as indication of a positive colony. The test was performed on two independent occasions in duplicate.

### 3.6. Ability to Form a Biofilm

Overnight cultures of each *L. sakei* CNTA 173 strain were diluted 1:100 (*v*/*v*) in fresh MRS medium. Then, 200 µL of this bacterial suspension was inoculated into each well of a 96-well polystyrene microtiter plate (Nunclon Delta Surface from Thermo Fisher Scientific, Wilmington, DE, USA) and the plates were incubated for 24 h at 30 °C for anaerobiosis. Afterward, the planktonic phase was removed, and the biofilms were washed twice with phosphate-buffered saline (PBS; 137 mM NaCl, 2.7 mM KCl, 10 mM Na_2_HPO_4_, 2 mM Na_2_HPO_4_ [pH 7.4]). Total biomass was quantified by performing the crystal violet staining assay, as described previously [55]. Briefly, after washing the biofilm with PBS, 0.2 mL of 0.1% (*w*/*v*) crystal violet was added to each well. After fifteen minutes, the excess crystal violet was removed by rinsing twice with water. The remaining dye was then solubilized by adding 33% (*v*/*v*) acetic acid, and the absorbance at 570 nm (A570) was measured in a microplate reader (Multiskan Ascent, Thermo Labsystems, Vantaa, Finland).

### 3.7. Qualitative Determination of Bile Salts Hydrolase Activity (BSH Activity)

Early stationary phase culture of *L. sakei* CNTA 173 was streaked on an MRS agar plate or an MRS agar plate supplemented with 0.5% (*w*/*v*) sodium salt of glycocholic, glycodeoxycholic, taurocholic, or taurodeoxycholic acids (Cymit Química) and 0.375 g/L CaCl2 (Sigma-Aldrich). Plates were kept in an anaerobic environment at 37 °C for 72 h. The white precipitates around microbial growth and the clearing of the medium were indicative of BSH activity [56].

### 3.8. Adhesion

#### 3.8.1. Bacteria and Growth Conditions

*E. coli* O157:H7 enterohemorrhagic strain DSM 19206 was obtained from DSMZ (Braunschweig, Germany). *L. sakei* were cultured in MRS broth at 30 °C in anaerobic conditions, and *E. coli* were cultured in TSB +YE broth (Scharlab S.L.) at 37 °C in aerobic atmosphere.

#### 3.8.2. Caco-2 Cell Culture

The Caco-2 human colon adenocarcinoma cell line (ACC 169) was obtained from DSMZ. Cells were routinely cultured in Dulbecco’s modified Eagle’s medium (DMEM 10569 Gibco high glucose + GLUTAMAX + sodium pyruvate, Thermo Fisher Scientific, Wilmington, DE, USA) with 20% foetal bovine serum (Fetal Bovine Serum, qualified, heat inactivated, Brazil, A310500064, Thermo Fisher Scientific) and 1% of penicillin–streptomycin (P0781, Sigma-Aldrich, Saint Louis, MI, USA).

The incubation was carried out at 37 °C in 5% CO_2_ atmosphere in a humidified incubator. For adhesion assay, Caco-2 cells were seeded at a concentration of 3 × 10^4^ cells/well in 24-well tissue culture plates (Costar 24-well Clear TC-treated Multiple Well Plates, Individually Wrapped, Sterile, Corning, NY, USA). After they reached confluence (ca. 1 week), cells were cultured for 1 week more to become fully differentiated. At least 96 h before the tests, cultured medium without antibiotics was used.

#### 3.8.3. Adhesion Assays on Caco-2 Cells

Bacterial adhesion tests on Caco-2 cells monolayer were carried out in 24-well tissue culture plates. *E. coli* and *L. sakei* from 16 h cultures in TSB + Ye and MRS broths, respectively, were washed once with distilled water with peptone 0.1% (Merck KGaA). DMEM was used for resuspending bacteria in appropriate dilutions for the different tests. After washing the Caco-2 cells twice with phosphate-buffered saline (DPBS without calcium or magnesium, pH = 7.3 (A314190094, Invitrogen Life Technologies, Carlsbad, CA, USA)), 0.5 mL of DMEM with bacterial suspension was added to each well and incubated for 60 min in atmosphere with 5% CO_2_ at 37 °C. Unattached bacteria were removed by 3-fold washing with DPBS without calcium or magnesium. To select microorganism concentration, three different concentrations around 1:1 to 1:100 relationship cells/bacteria of *L. sakei* were tested (from ca. 4 × 10^5^ to 9 × 10^7^ CFU/well) in three independent experiments). Subsequently, the similar ratio of *L. sakei* was used for *E. coli*. In competition assays, *L. sakei* were added simultaneously with *E. coli* [57]. To test possible exclusion and displacement of *E. coli* by *L. sakei* or vice versa, the incubation of Caco-2 cells with one strain was followed by washing of unattached cells, addition of the second strain, and another 60 min of incubation [24]. One different concentration of *L. sakei* (2.6 × 10^6^ ± 1.1 × 10^6^ CFU/well) and *E. coli* (9.2 × 10^6^ ± 6.5 × 10^6^ CFU/well) was tested in three independent experiments.

The exact number of viable *L. sakei* and *E. coli* in the bacteria suspensions used in the assays was determined by plate counting. In each experiment, plate counting was performed on MRS and Violet Red Bile Dextrose (VRBD) agar media (Merck KGaA), for *L. sakei* and *E. coli*, respectively. MRS agar plates were incubated for 2 days in anaerobiosis and VRBD agar plates for 24 h in aerobiosis, both at 37 °C. Caco-2 cells were separated off the plate by 0.05% Trypsin-EDTA (Sigma Chemical Co., St. Louis, MO, USA) for 5 min, and adherent *L. sakei* and *E. coli* were enumerated (CFU/well) in the same medium and conditions of bacteria suspensions.

### 3.9. Immunomodulation and Cell Viability

#### 3.9.1. Bacterial Culture

*L. sakei* were cultured in MRS broth at 30 °C in microaerophilic atmosphere in an anaerobic incubator, then culture was centrifugated, washed with DPBS without calcium and magnesium and resuspended in glycerol 30%. Dilutions with cells medium were performed to obtain a final concentration in well of 2 × 10^6^ UFC.

#### 3.9.2. Cell Culture and Maintenance

All cell lines were cultured at 37 °C in a humidified CO_2_ incubator with 5% CO_2_. RAW 264.7 murine cell line was kindly provided by Soria Natural (ATCC). Cells were maintained in DMEM (10569, Gibco, Life Technologies, Carlsbad, CA, USA) supplemented with 10% heat-inactivated serum (10500064, Gibco) without antibiotics. Passages were performed by gently detaching cells with a cell scraper. HT-29 cells were purchased from the DSMZ and cultured in McCoy’s medium (5A 26600-023, Gibco) and supplemented with 10% FBS (heat inactivated, 10500064 Gibco). After reaching confluency, cells were detached using 0.25% Trypsin-EDTA (25200056, Gibco). Cell debris were eliminated by washing with DPBS without calcium or magnesium (Gibco, 14190094)

#### 3.9.3. Cell Viability Assays

Cell viability was assessed using the neutral red assay as previously described [58]. In short, RAW 264.7 mouse macrophage cells were seeded at a concentration of 34,000 cells per well in 96-well plates, and then cultured for 24 h. *L. sakei* were cultured in MRS broth (Merck KGaA) at 30 °C in microaerophilic atmosphere in an anaerobic incubator, then, the culture was centrifuged, washed with DPBS without calcium and magnesium, and resuspended in glycerol 30%. Dilutions with cells medium were prepared to obtain a final concentration in well of 2 × 10^6^ UFC. Then, cell pellet of bacteria was added for the specified time and stimulated with 1 µg/mL lipopolysaccharide (LPS). Afterwards, supernatants were collected for cytokine quantification, and cells were stained with neutral red. Fluorescence was quantified with a SynergyHTX plate reader (Biotek, Winsooki, VT, USA) using 530 nm excitation and 630 nm emission filters. Data were normalised to the negative control (untreated cells). The same protocol was used for the cell line HT-29, where 17,000 cells were seeded per well and cultured for 48 h prior to performing the assay.

#### 3.9.4. Cytokine Quantification

After stablishing cell viability, RAW 264.7 cells were seeded in 96-well plates at a concentration of 34,000 cells per well, then cultured for 24 h. After reaching confluency, cells were pre-treated with the indicated bacterial pellets for 3 h, and then stimulated with 1 µg/mL LPS for 18 h, without changing the media. Cell supernatants were collected and analysed by ELISA KIT for IL-6, IL-8, IL-10, and TNF-α according to the manufacturer’s instructions. For HT-29 cell line, cells were seeded in 96-well plates in a concentration of 17,000 cells/well, then incubated for 48 h. Afterwards, cells were treated for 3 h with *L. sakei* and then stimulated with 1 µg/mL LPS for 18 h. Supernatants were collected for IL-8 quantification by ELISA.

### 3.10. Statistical Analysis of In Vitro Probiotic Characterization

The number of biological replicates is stated for each experiment in the figure legend, being *n* = 3 at minimum. First, distribution of residuals was evaluated using the Shapiro–Wilk test and others. Then, homogeneity of variance was assessed. Finally, one-way ANOVA or Kruskal–Wallis statistics were performed and specified in the figure legend.

### 3.11. L. sakei CNTA 173 In Vivo Activities in C. elegans

#### 3.11.1. Nematode Culture and Experimental Design

The C. elegans strains were obtained from the Caenorhabditis Genetics Centre (CGC, University of Minnesota, Minneapolis, MN, USA). Wild-type N2 Bristol, daf-16 (mu86, CF1038) mutant strain, daf-22 mutant strain (RB859 Y57A10C.6(ok693) I), acox-1 mutant strain (VC1785 F08A8.1(ok2257) I), and maoc-1 mutant strain (VS18 (hj13) II), were cultured on nematode growth medium (NGM) at 20 °C and *E. coli* OP50 (grown in LB Broth Lennox at 37 °C) as normal nematode diet. All assays were performed in 6-well plates in quadruplicate with 4 mL of NGM or glucose-supplemented (10 mM) NGM (NGMg). For the gene expression analyses, six replicates were used for each condition. Orlistat-supplemented plates (1.5 mg/mL, Sigma Aldrich) were used as positive controls of fat reduction. The probiotics were embedded in the NGM at the dose of 3 × 10^5^ CFU/mL (D1) and 3 × 10^6^ CFU/mL (D2) and compared with sterile water for negative control (NGM group). Then, plates were left overnight to solidify in a dry and dark place. After two days, 150 µL of overnight cultured *E. coli* OP50 were spread on the plates and left at room temperature until dry. In all assays, nematodes were age-synchronized by standard hypochlorite treatment. Eggs were then hatched overnight in M9 medium at 20 °C. Approximately 300 L1 larvae were transferred to each NGM well and grown for two days until L4 adult phase was reached, at which time the staining and imaging experiments were performed.

#### 3.11.2. Nile Red and DHE Staining

Red Nile natural dye (#N3013, Sigma-Aldrich, St. Louis, MO, USA), which binds free lipids, was used to compare via fluorescence microscopy differences in fat accumulation between treatments [59]. L4 stage nematodes grown in NGM or NGMg with and without *L. sakei* CNTA 173 (4 replicates per condition) were collected in 1.5 mL tubes and washed with PBST (0.01% of Triton X-100 in phosphate-buffered saline) 3 times. Then, nematodes were put on ice for 15 min and fixed in 40% isopropanol for 3 min. Staining was carried out by adding 150 μL of Nile red solution (3 μg/mL) per tube and incubating (30 min) with moderate shaking at room temperature in the dark. Finally, nematodes were washed with PBTS and mounted on microscopy slides containing a solidified droplet of agarose 2%.

Moreover, we used the fluorescent dye dihydroethidium (DHE; Dihydroethidium BioReagent, ≥95% (HPCE), Sigma-Aldrich) to quantify oxygen reactive species (ROS) levels in vivo [60]: 250 synchronized L1 larvae/well were transferred onto NGMg plates with the embedded probiotics (or water in control group) and allowed to grow until L4 adulthood. Then, nematodes were harvested in 1.5 mL tubes, washed with PBST, and incubated in 3 μM DHE solution (in PBS) for 90 min. Finally, nematodes were washed with PBTS and mounted on microscopy plates with a solidified droplet of agarose 2% containing sodium azide at 1%.

#### 3.11.3. Image Acquisition and Quantification

In all conditions tested, an approximate number of 200 nematodes were fixed after processing in microscopy slides. Fluorescent images of Nile red and DHE were captured as previously described [60].

#### 3.11.4. *C. elegans* Lifespan Analysis

Lifespan analysis was performed as previously described [61]. Briefly, the synchronized L1 larvae were transferred to NGM or NGMg plates with the embedded probiotics or water as control in quadruplicates and grown for two days until L4 stage. Then, an approximate number of 40 nematodes/replicate were transferred to identical plates containing 40 µM of 5-fluoro-2-deoxyuridine (FUDR, #856657, Sigma-Aldrich). Live nematodes were counted daily, and dead ones, which failed to respond to gentle touch, were removed using a sterilized silver wire.

#### 3.11.5. Egg Laying

We ensured the correct development of the nematodes under the effects of probiotic treatment after three days of growth from the L1 larvae stage to L4 adulthood. On day 3, the presence of eggs and L1 larvae were checked in NGM and NGMg plates with and without *L. sakei* CNTA 173 treatments. The images were taken at 40× magnification using a Nikon SMZ18 stereomicroscope equipped with a Nikon DS-Fi1C high-definition colour camera (Nikon Corporation, Tokyo, Japan).

#### 3.11.6. *C. elegans* RNA Extraction and Quantitative PCR Analysis

RNA extraction and qPCR analyses were carried out as reported previously [47]. Briefly, L4 nematodes previously grown in the presence of the probiotic or water were treated with TRIzol^®^ RNA isolation reagent (Thermo Fisher Scientific Inc.) to extract total RNA. The concentration and purity of RNA were determined at 260/280 nm using a NanoDrop ND-1000 spectrophotometer (Thermo Fisher Scientific). Subsequently, 500 ng of RNA were treated with DNase I (DNase I-RNase free, Invitrogen Life Technologies) according to the standard protocol and were reverse-transcribed using 200 IU of M-MLV-RT (Invitrogen Life Technologies) in the presence of 40 IU of recombinant RNAsin^®^ ribonuclease inhibitor (Promega, Madison, WI, USA), with an incubation of 10 min at 25 °C, 50 min at 37 °C, and 15 min at 70 °C.

Gene expression analyses were performed by quantitative-real time PCR (qPCR) in triplicate using the TaqMan™ Universal PCR master mix with specific probes from Applied Biosystems™ (Thermo Fisher Scientific, Foster City, CA, USA) (TaqMan™ Gene Expression Assays, ESI Table 1) and IDT Technologies (Integrated DNA Technologies Inc., Coralville, IA, USA, ESI Table 2), using a CFX384 Touch™ Real-Time PCR Detection System (Bio-Rad Laboratories, Hercules, CA, USA). All assays were analysed in triplicate. The expression levels of each gene were normalized using the expression of Peroxisomal Membrane Protein-related 3 (pmp-3) as reference housekeeping gene. Gene expression differences between *L. sakei*-treated and untreated samples were quantified using the relative quantification 2^−ΔΔCt^ method (14).

#### 3.11.7. Statistical Analysis of *C. elegans* Determinations

Body fat estimation (Nile red) and ROS quantification (DHE) were evaluated between groups by Student’s *t*-test (2 samples) or ANOVA test, when testing various dosages, followed by multiple comparison (Fisher’s protected Least Significant Difference, LSD) tests. The differences in lifespan among groups were tested by performing log-rank (Mantel–Cox) test between treatment and control (NGM) groups. qPCR data were analysed by two-way ANOVA (main effects: *L. sakei*, glucose, and their interaction), followed by a Student’s *t*-test when interactions were found. Tests were performed using either StataSE v12 software (StataCorp LLC, College Station, TX, USA) or GraphPad Prism 8.0 (GraphPad Software, Inc., San Diego, CA, USA).

## 4. Conclusions

This work presents the probiotic properties of a novel strain of *L. sakei* (CNTA 173), demonstrating its sensitivity to the entire spectrum of antibiotics analysed, gastric and intestinal resistance in vitro, β-galactosidase activity, and the ability to form biofilm and to produce acetic acid. Moreover, this novel strain *L. sakei* CNTA 173 is able to reduce the adhesion of the pathogenic *E. coli* O157:H7 in Caco-2 cells and exerts an immunomodulatory capacity in vitro without affecting cell viability. Functional in vivo studies demonstrate that *L. sakei* CNTA 173 is able to counteract the obesogenic effects of glucose exposure in *C. elegans* by reducing fat accumulation, enhancing the oxidative stress response, and extending lifespan by directly regulating the carbohydrate and lipid metabolism-related genes *acox-1*, *maoc-1*, and *daf-16*. Although further studies in mammals are necessary to confirm its physiological effect, our results open the possibility of using this new strain as a probiotic candidate for the prevention and/or treatment of metabolic diseases associated with excess of adiposity or oxidative stress, such as obesity or type 2 diabetes.

## 5. Patents

The works reported in this manuscript have resulted in the registration of the patent EP24383384, entitled: LATILACTOBACILLUS SAKEI STRAIN AND USES THEREOF.

## Figures and Tables

**Table 1:** Antibiotic susceptibilities of *L. sakei* CNTA 173 (mg/L) *.

Strain			MIC Values Against Antibiotics (mg/L)					
	Gm	Km	Sm	Tc	Cl	Cm	Am	Em
*L. sakei* CNTA 173	4	16	64–16	2	0.062–0.032	2	4	0.125
MIC cut-off values *	≤16	≤64	≤64	≤8	≤1	≤4	≤4	≤1

Gm, gentamycin; Km, kanamycin; Sm, streptomycin; Tc, tetracycline; Cl, clindamycin; Cm, chloramphenicol; Am, ampicillin; Em, erythromycin. * Established MIC breakpoint values for *Lactobacillus* facultative heterofermentative (EFSA). Those values indicating susceptibility to each specific antibiotic are written in green.

**Table 2:** Measurements of acetic acid in the medium after growth of *L. sakei* CNTA 173.

Medium	Concentration (g/L)
Defined medium without adding carbon source	1.46 ± 0.01
Defined medium with glucose (2%)	0.40 ± 0.04
Defined medium with Synergy 1 (2%)	1.06 ± 0.10
Defined medium with P95 (2%)	1.33 ± 0.03

## Data Availability

Data contained within the article.

## Supplementary Materials

The following supporting information can be downloaded at: www.mdpi.com/article/10.3390/ijms26073286/s1.
