# Supplementary material for: Anti-Obesity Properties of a Novel Probiotic Strain of Latilactobacillus sakei CNTA 173 in Caenorhabditis elegans
Source: Int J Mol Sci. 2025 Apr 1;26(7):3286. doi: 10.3390/ijms26073286 (PMC11989871; doi:10.3390/ijms26073286)
Supplement: Supplementary file 1 [file ijms-26-03286-s001.zip › ijms-3522506-supplementary.pdf]

Supplementary information

Supplementary tables

Table S1: ANI values when comparing the genome of *Latilactobacillus* sp. CNTA173 with all available genomes of the type of strain *Latilactobacillus sakei*.

Reference type strain	Accession number (GenBank)	ANI (%)
Latilactobacillus sakei subsp. sakei NBRC 15893(T)	GCA_007989165.1	98,738
Latilactobacillus sakei subsp. sakei ATCC 15521(T)	GCA_004354475.1	97,516
Latilactobacillus sakei subsp. sakei DSM 20017(T)	GCA_002370375.1	97,485

Table S2: Strains closest to *Latilactobacillus* sp. CNTA 173 according to the ANI index.

Strain	Accession number (GenBank)	ANI (%)
Latilactobacillus sakei CECT 9267	GCA_900290175.1	99,544
Latilactobacillus sakei WiKim0095	GCA_024022895.1	99,373
Latilactobacillus sakei WiKim0095	GCA_003627235.1	99,036
Latilactobacillus sakei JD10	GCA_022559145.1	98,960

Table S3: Main results of genomic analysis. The genome size was calculated with QUAST. The degree of completeness and contamination was calculated with CheckM.

Strain	Size	Completeness	Contamination	GC %
CNTA 173	1.28 Mbp	99.18%	0.63%	40.97

Table S4: Growth parameters of *L. sakei* CNTA 173.

Parameters	*L. sakei* CNTA 173
Initial bacteria count (**A**) (Log CFU/mL)	6.26
Maximum growth rate (**B**) (hours $^{-1}$)	0.61
Difference between the initial bacteria count and the maximum bacteria count (**C**) (Log CFU/mL)	2.28
Time required by microorganism to achieve the maximum growth rate (**M**) (hours)	6.13
Lag time (LPD) (hours)	4.49
R value	0.99
RMSE value	0.11

Supplementary figures

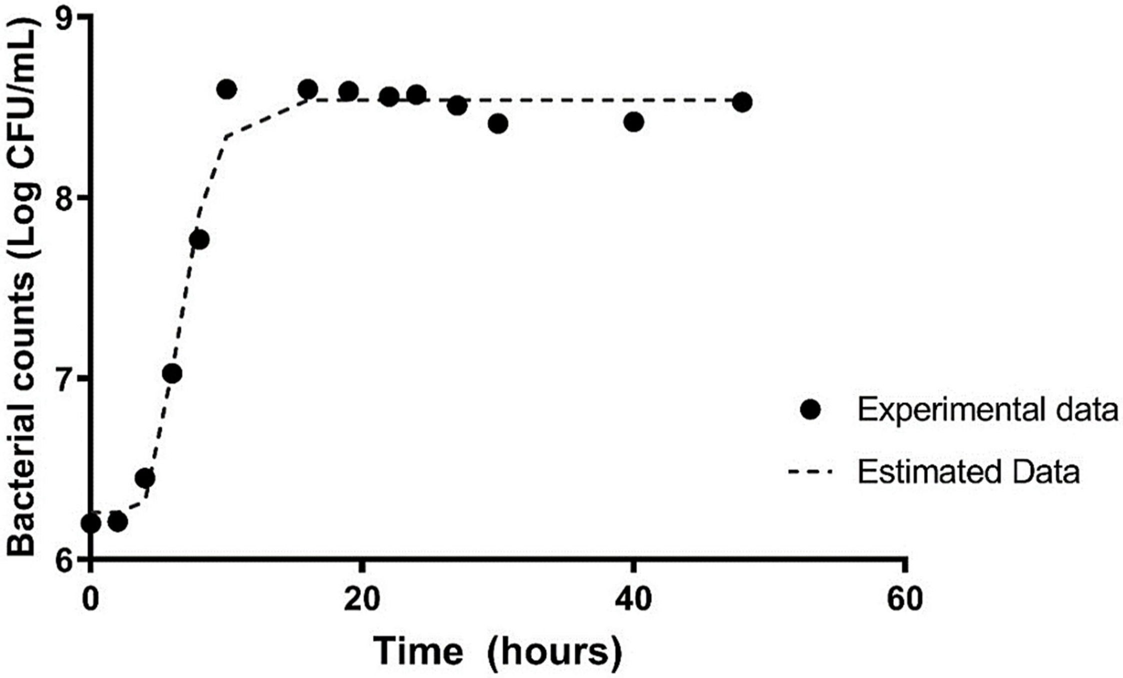

Figure S1. Growth curve of *L. sakei* CNTA 173 through the incubation time at 30ºC in MRS Broth. Experimental values and estimation with the Gompertz model.

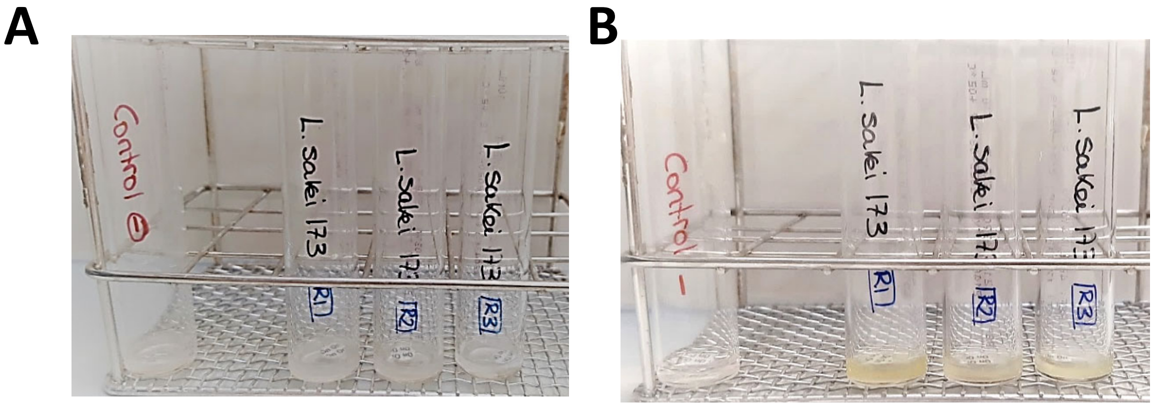

Figure S2: A) Tubes with bacterial culture of *L. sakei* CNTA 173 in contact with the ONPG disk at time 0, exhibiting a complete lack of coloration. B) Tubes with bacterial culture of *L. sakei* CNTA 173 in contact with the ONPG disk at 5 hours, exhibiting a change in color.

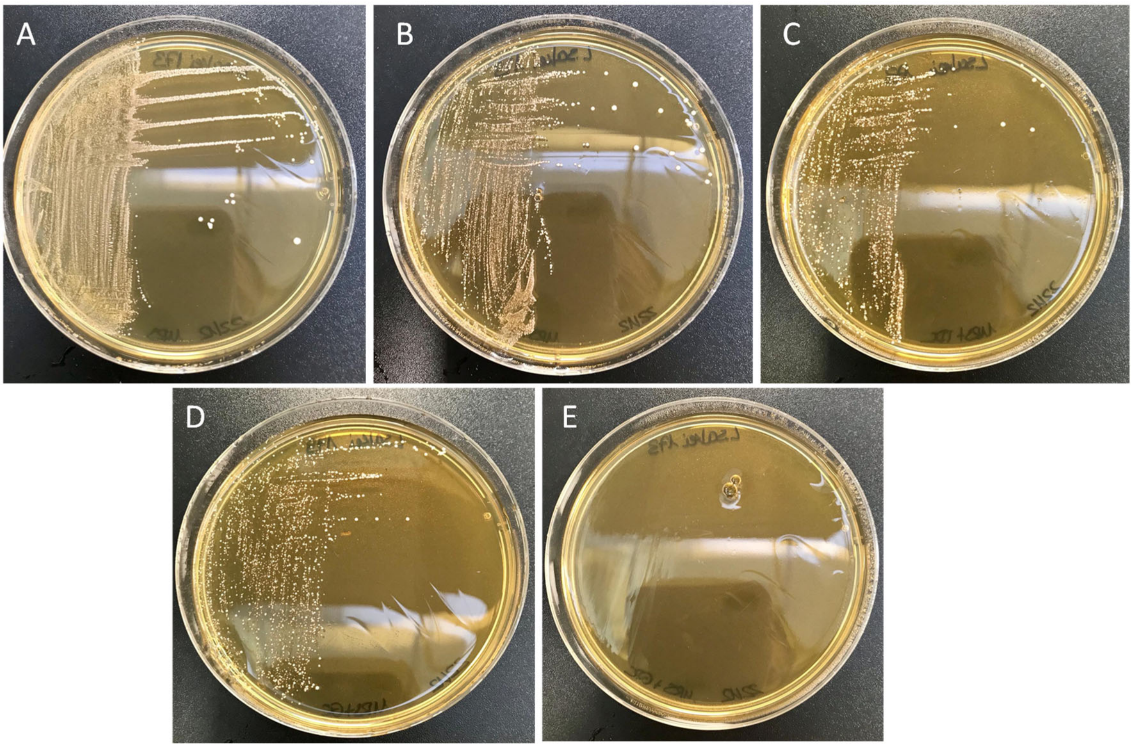

Figure S3: Morphology of *Lactobacillus sakei* CNTA 173 colonies in MRS agar plate (A) or in MRS agar plate supplemented with 0.5% sodium salt of taurocholic (B), taurodeoxycholic (C), glycocholic (D) or glycodeoxycholic acids (E).

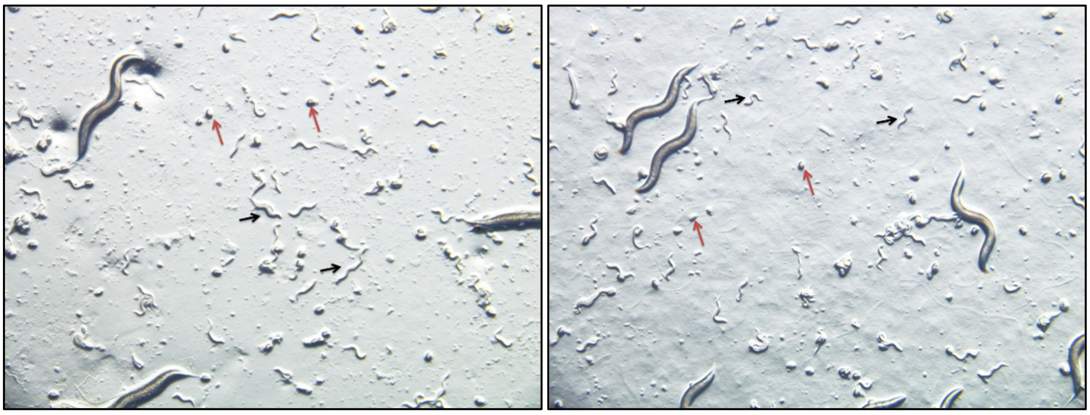

Figure S4: Microscope observations of laid eggs (red arrow) and L1 stage larvae (black arrow) from de *L. sakei* CNTA 173-treated group and the NGM group (right).
